# Stability at a Cost: Evaluating Postoperative Complications in Bioabsorbable vs. Metallic Syndesmotic Fixation: A Systematic Review

**DOI:** 10.7759/cureus.102907

**Published:** 2026-02-03

**Authors:** Andy Suarez, Leon Liu, Taylor Checkley, Samir Sakaria, Alejandra Rinaldi, Chrisitian Palacios, Adrian Alepuz, Micah Ngatuvai, Gary Schwartz

**Affiliations:** 1 Health Professions Division, Nova Southeastern University Dr. Kiran C. Patel College of Allopathic Medicine, Fort Lauderdale, USA; 2 Health Sciences Department, Nova Southeastern University Dr. Kiran C. Patel College of Osteopathic Medicine, Fort Lauderdale, USA; 3 Orthopaedic Surgery Department, Larkin Community Hospital, Miami, USA; 4 Orthopaedic Surgery Department, Texas Tech University Health Sciences Center, El Paso, USA

**Keywords:** bioabsorbable screws, distal tibiofibular syndesmotic injury, meta-analysis, metallic screws, syndesmotic fixation

## Abstract

The use of bioabsorbable screws for syndesmotic fixation in complex ankle fractures has become increasingly popular, as they may eliminate the need for secondary surgeries for implant removal. However, their efficacy when compared against metallic screws is unclear, with some persisting concerns of higher complication rates compared to the traditional metallic option. A systematic search was conducted using PubMed and Google Scholar, yielding 332 studies. After title and abstract screening followed by full-text review, eligible randomized controlled trials comparing bioabsorbable versus metallic screw fixation in ankle syndesmotic injuries were included. The total rate of postoperative complications was primarily assessed. Risk ratios (RR) and 95% confidence intervals (CIs) were calculated. Four studies met the inclusion criteria for the meta-analysis, encompassing 257 patients. The pooled data demonstrated a significantly higher risk of complications with bioabsorbable screw fixation, including revision surgery, infection, and symptomatic hardware removal, compared to metallic screws (RR 3.35, 95% CI 1.75-6.42, p = 0.0003). Range-of-motion outcomes varied across studies but showed no clear superiority of either fixation method. The increased complication rate with bioabsorbable screws raises concerns about routine use in syndesmotic fixation. Although previous studies have suggested comparable functional outcomes and fewer reoperations due to implant removal, the current data underscore the importance of prioritizing safety and reliability. Given the risk of inflammatory reactions and hardware failure seen in bioabsorbable groups, metallic screws remain the more dependable option. Future research should focus on improving the biomechanical and biocompatibility profiles of absorbable materials before they are widely adopted in clinical use.

## Introduction and background

Distal tibiofibular syndesmotic disruptions, commonly referred to as syndesmotic injuries, have an annual incidence of 15 per 100,000 individuals [1]. These present either as an isolated syndesmotic rupture or in conjunction with an ankle fracture, which accounts for 10% of cases [1]. Cornu et al. detailed that syndesmotic injuries are found in 40% of all grade one and grade two ankle sprains and 20% of grade three sprains [2]. Syndesmotic injuries are most commonly associated with an external rotation mechanism of injury [3,4]. When accompanied by ankle fractures, these injuries are frequently classified as either Weber C with a Lauge-Hansen pronation external rotation pattern or Weber B with a Lauge-Hansen supination external rotation pattern [5-8].

Traditional operative management of ankle fractures occurring with syndesmotic ruptures consists of internal fixation with metallic screws and plates, allowing for the correction of joint alignment alongside a temporarily restricted range of motion from the placement of a syndesmotic screw, which limits tibiofibular translation and may lead to irritation [9,10]. As a result, this surgical approach is often followed by another procedure for screw removal once the syndesmosis has healed [9,11]. Although removal is not always indicated, certain cases recommend hardware removal between eight and 12 weeks after the initial procedure [12].

While the second procedure helps restore normal ankle biomechanics, it also carries additional risks, including an increased possibility of infection, prolonged recovery time, and increased financial burden for the patient [9,11,13]. Consequently, there has been an increasing number of developments focused on stabilizing distal tibiofibular syndesmotic disruptions without requiring a follow-up procedure [1,13]. Such surgical approaches include the use of bioabsorbable screws or suture anchors [7,14].

Three different bioabsorbable screws used in the fixation of complex ankle fractures with syndesmotic disruption are described in the literature: polyglycolic acid (PGA), polylactic acid (PLA), and polylevolactic acid (PLLA) [9,13]. Though the concept of bioabsorbable implants was devised to circumvent a second procedure for screw removal, a removal procedure may still be indicated in certain cases [13].

Other concerns regarding the comparison of the two screw materials persist. Hovis et al. reported that bioabsorbable implants are mechanically inferior to traditional metallic implants in internal fixation procedures [15]. Similarly, Gaiarsa et al. noted that metallic implants can sustain more force than bioabsorbable implants [16]. However, an analysis of syndesmosis fixation using bioabsorbable implants demonstrated sufficient mechanical strength [9,17]. Nonetheless, despite a few comparative studies, there remains uncertainty regarding whether bioabsorbable or metallic screws represent the more appropriate clinical and functional treatment for syndesmotic ruptures associated with complex ankle fractures.

This meta-analysis was performed to compare the efficacy of bioabsorbable screws versus metallic screws in distal tibiofibular syndesmotic disruptions by evaluating the overall rates of postoperative complications associated with each respective surgical technique. With this close comparison, the risk-reward of using bioabsorbable screws instead of traditional metallic screws may be better quantified.

This research project was previously presented as a rapid-fire session at the Clinical Orthopaedic Society's 113th annual conference on September 4, 2025, and as a poster presentation at the Society of Military Orthopaedic Surgeons' 67th annual meeting on December 12, 2025.

## Review

Methods

Search Strategy and Selection Criteria

In March 2025, a systematic search was conducted on PubMed and Google Scholar for studies comparing the functional outcomes of patients treated with bioabsorbable and metallic screw fixation. Included studies were restricted to patients at least 18 years of age. Case reports, retrospective studies, cadaver studies, animal studies, and systematic reviews were excluded, and duplicate patient cohorts were omitted. The inclusion criteria designated 3 prospective randomized control trials and a comparative study that were used in this analysis (Table 1). No quasi-randomization of any of the references was mentioned. This review was conducted in accordance with Preferred Reporting Items for Systematic Reviews and Meta-Analyses (PRISMA) guidelines.

**Table 1:** Selected studies

Study	Year	Design	Sample Size	Intervention/Exposure	Outcome Measures	Key Results	Location
Thordarson et al. [9]	2001	Prospective randomized trial	34	Bioabsorbable screw (PLA); metallic stainless-steel screw	Syndesmotic stability; hardware removal; functional outcomes	No significant functional differences; Bioabsorbable avoided screw removal; Comparable complications	USA
Gaiarsa et al. [16]	2015	Comparative study	40	Bioabsorbable implants; metallic implants	Radiographic outcomes; functional recovery; complications	Similar outcomes; Metallic implants had more soft-tissue irritation; Union rates similar	Brazil
Kaukonen et al. [17]	2005	Randomized controlled trial	38	Bioabsorbable screw (PLA); metallic screw	Malreduction; screw removal; functional outcomes	No difference in reduction; Bioabsorbable avoided screw removal; Similar functional outcomes	Finland
Sun et al. [18]	2014	Prospective randomized trial	168	Absorbable screw (PLLA); metallic screw	AOFAS scores; radiographic reduction; complications	Absorbable screws had better mid-term scores; Lower reoperation; Similar radiographic outcomes	China

Study Selection

Following the search through the databases, studies were uploaded to Covidence (Alfred Health, Melbourne, Australia). The data extraction tool was used, and duplicates were automatically excluded. Two independent reviewers screened the articles based on title and abstract. Studies that did not include the outcomes of interest or follow the appropriate study design were excluded. Studies that remained underwent full-text screening by two independent reviewers using the predetermined inclusion and exclusion criteria. Each reviewer worked independently, and article selection was blinded. Discrepancies in article selection were resolved through discussion with a third reviewer.

Data Extraction and Risk of Bias

Data extraction was performed by two independent reviewers utilizing templates provided by Covidence. The templates were used to collect data regarding the background of the article, methods used, population characteristics, interventions, and outcomes. Some studies also reported outcomes specific to their unique research objectives; however, those outcomes were not included in this meta-analysis.

Risk of Bias was assessed using a meta-analysis tool, Review Manager (RevMan) version 5.4.1 (Nordic Cochrane Centre, The Cochrane Collaboration 2020, Copenhagen, Denmark). Various parameters of each study were scored by two individual reviewers using a scale of low to high likelihood of bias. Parameters scored involved aspects of study design, such as blinding, selective reporting, and random sequence generation.

Data Analysis

Extracted data were pooled and analyzed using RevMan. Risk ratios were estimated, and the Mantel-Haenszel statistical method was applied. A fixed effect model was also applied, and point estimates were calculated with their corresponding 95% confidence interval (CI). All tests were given a statistical significance threshold of 0.05, and heterogeneity was assessed by calculating the I² index, with values >50% representing moderate to high heterogeneity.

Results

**Figure 1:**
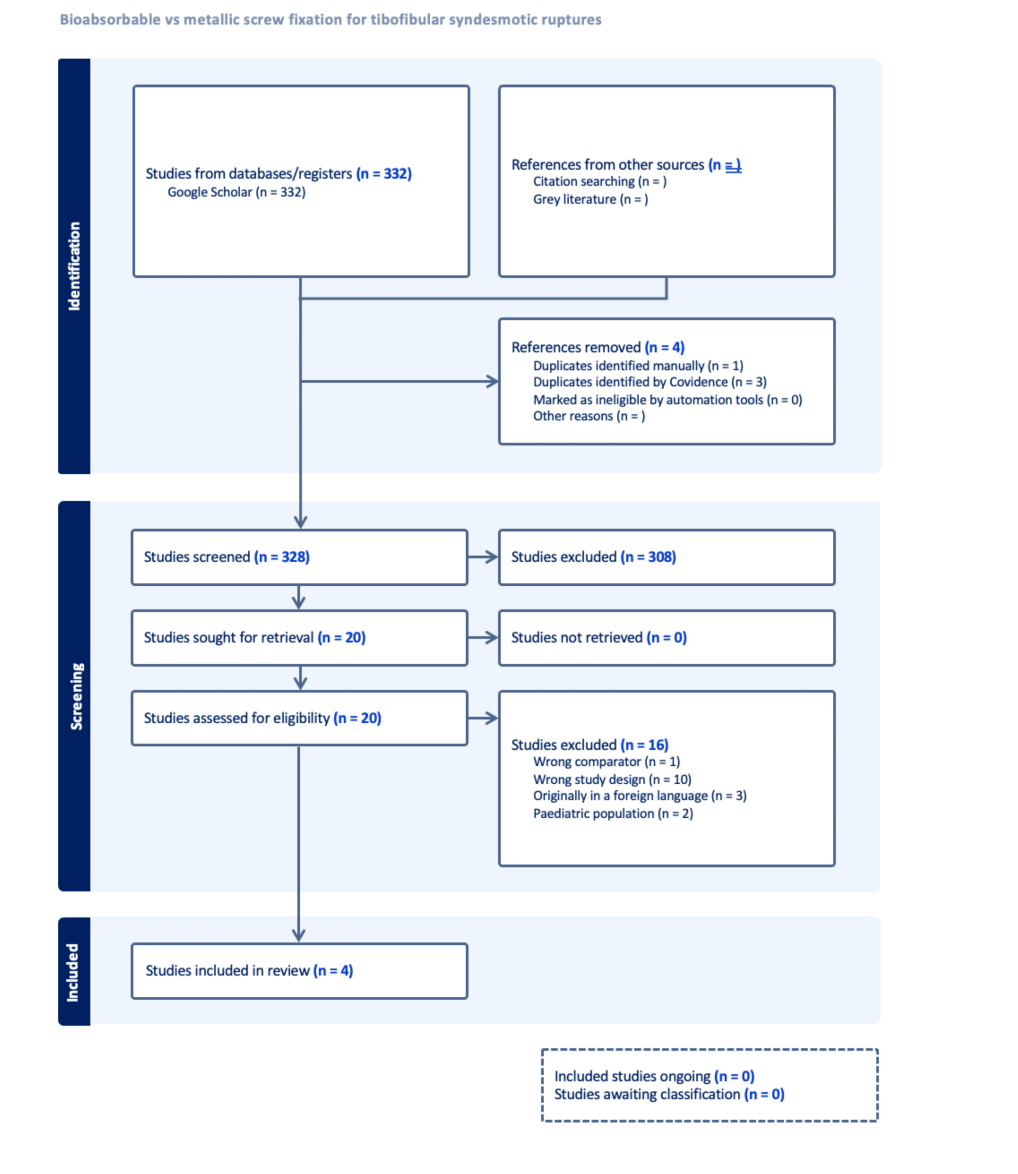
Preferred Reporting Items for Systematic Reviews and Meta-Analyses (PRISMA) flow diagram

After duplicated studies were removed, 328 studies underwent abstract and title screening. After the resulting studies underwent full-text screening, a total of four studies were eligible for inclusion in the final data analysis of this meta-analysis (Figure 1).

Outcomes

**Figure 2:**
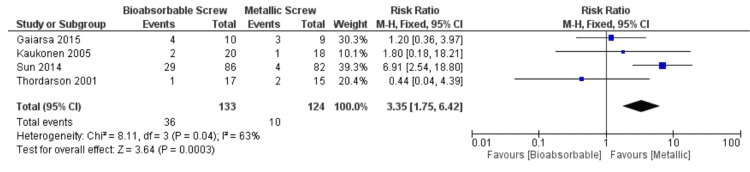
Total complications for bioabsorbable vs. metallic screws References: Thordarson et al. [9], Gaiarsa et al. [16], Kaukonen et al.[17], Sun et al. [18]

The analyses of the data pooled from the four included studies showed a significant difference in the total complications experienced by patients who received bioabsorbable fixation and those who received metallic fixation. Complications included revision surgery, infection, and symptomatic hardware removal. Data from a total of 257 patients revealed a RR of 3.35, favoring the use of metallic screw fixation in the repair of ankle syndesmotic injuries (RR 3.35, CI 95%, 1.75 to 6.42, p = 0.0003; Figure 2). Total heterogeneity of the study (I²) was 63%.

A few studies also reported on patients’ range of motion. Kaukonen reported a lower mean angle of dorsiflexion in bioabsorbable screw fixation (22.2 degrees compared to 25.2 degrees in the metallic group) and a slightly greater mean angle of plantarflexion in the bioabsorbable screw fixation group (45.9 degrees compared to 45.4 degrees in the metallic screw group). Conversely, Sun reported greater mean angles of dorsiflexion and plantarflexion in the bioabsorbable screw group (22.0 and 40.0 degrees, respectively, compared to 18 and 35 degrees in the metallic screw group).

Risk of Bias

**Figure 3:**
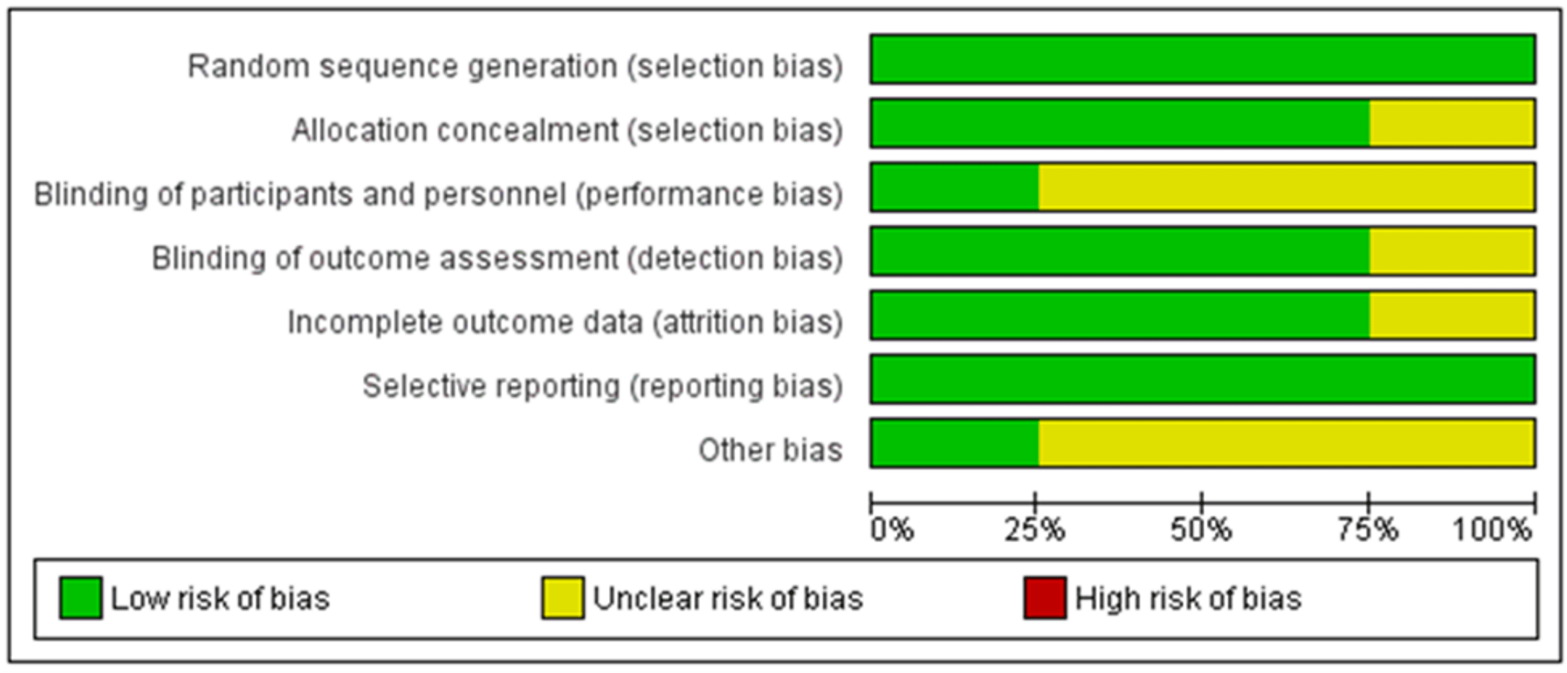
Risk of bias graph

**Figure 4:**
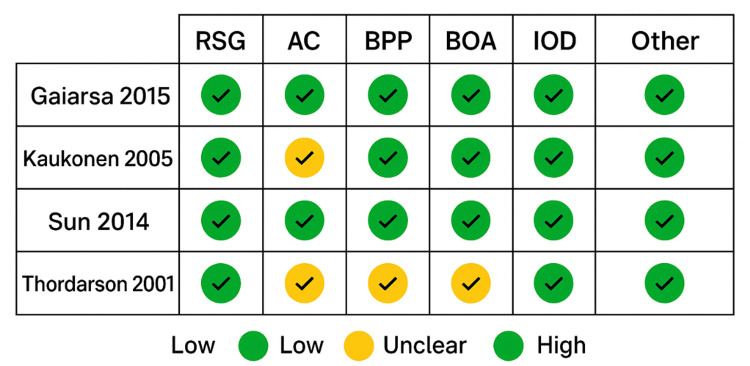
Risk of bias summary AC, allocation concealment; BPP, blinding of participants and personnel; IOD, incomplete outcome data; RSG, random sequence generation; SR, selective reporting References: Thordarson et al. [9], Gaiarsa et al. [16], Kaukonen et al.[17], Sun et al. [18]

Risk of bias was assessed for all four included studies. The primary source of bias among our included studies was blinding of participants and personnel, consistent with the methodology and hypothesis of the studies revolving around a surgical intervention. A few articles also had unclear bias in the blinding of outcome assessment, allocation concealment, and completion of outcome data (Figures 3-4).

Discussion

This meta-analysis found that bioabsorbable screws are associated with a significantly higher rate of postoperative complications compared with metallic screws for fixation of distal tibiofibular syndesmotic injuries. These complications included the need for revision surgery, infection, and symptomatic hardware removal. The findings align with prior reports indicating that bioabsorbable implants possess weaker mechanical properties compared to metallic constructs, raising concerns regarding their long-term performance in load-bearing applications.

Although earlier studies by Thordarson et al. and Kaukonen et al. [9,17] reported comparable functional outcomes between bioabsorbable and metallic screws, the current analysis emphasizes safety rather than functional range. The range of motion findings were inconsistent. Kaukonen et al. [17] noted superior dorsiflexion with metallic screws, while Sun et al. reported greater dorsiflexion and plantarflexion with bioabsorbable implants [18]. However, the small number of studies assessing functional outcomes limits the strength of these observations. Importantly, the significantly higher complication rate associated with bioabsorbable implants suggests that any potential benefits in motion recovery are outweighed by increased risks.

Beyond screw fixation, suture-button devices have been introduced as an alternative for syndesmotic stabilization. These systems allow controlled micromotion between the tibia and fibula, reducing screw breakage and eliminating the need for routine hardware removal. Studies have shown that suture-button fixation can maintain anatomical alignment and lower malreduction rates compared with screws, though the devices carry higher initial costs [19].

While bioabsorbable screws theoretically eliminate a second operation for hardware removal, their elevated complication rate may offset this advantage. Costs related to managing infections, symptomatic implants, and revision procedures can exceed the savings of a single-stage approach. Thus, both economic and clinical considerations must be weighed when selecting fixation methods.

This study has several limitations. The small number of included trials (n = 4) and total patient sample (n = 257) reduces statistical power and limits subgroup analysis. Differences in surgical technique, implant material, and complication definitions also complicate direct comparison. Functional outcomes were inconsistently reported and not standardized across studies, restricting interpretation. Furthermore, the lack of blinding introduces potential performance and detection bias, and none of the included studies adjusted for confounders such as patient comorbidities or fracture severity. High risk of blinding was present due to the impracticality of complete blinding of patients and operating room personnel in surgical trials. This may have reduced the external validity of the study and contributed to heterogeneity. 

Additionally, newer materials such as magnesium alloys [20] and polylactic-co-glycolic acid (PLGA) blends were not represented in the included trials but are being evaluated for improved performance and biocompatibility. Larger multicenter randomized studies with standardized definitions, extended follow-up, and stratification by implant type are warranted. Despite these limitations, the consistent findings across available trials support the conclusion that metallic screws remain the more reliable and safer option for syndesmotic fixation in ankle fractures.

## Conclusions

This meta-analysis establishes that bioabsorbable screws are associated with a significantly increased incidence of postoperative complications compared to metallic screws in the surgical management of distal tibiofibular syndesmotic injuries. Despite prior reports of comparable functional outcomes, the elevated risk of infection, revision surgery, and symptomatic hardware removal underscores the safety profile of metallic implants. Until further improvements in the mechanical strength and biocompatibility of bioabsorbable materials are made, metallic screws should remain the standard of care for syndesmotic fixation.

## References

[1] Stein G, Eichler C, Ettmann L, Koebke J, Müller LP, Thelen U, Skouras E (2012). Tibiofibular screw fixation for syndesmotic ruptures: a biomechanical analysis. Surg Radiol Anat.

[2] Cornu O, Manon J, Tribak K, Putineanu D (2021). Traumatic injuries of the distal tibiofibular syndesmosis. Orthop Traumatol Surg Res.

[3] Yuen CP, Lui TH (2017). Distal tibiofibular syndesmosis: anatomy, biomechanics, injury, and management. Open Orthop J.

[4] Hermans JJ, Beumer A, Hop WC, Moonen AF, Ginai AZ (2012). Tibiofibular syndesmosis in acute ankle fractures: additional value of an oblique MR image plane. Skeletal Radiol.

[5] Yasui Y, Hannon CP, Seow D, Kennedy JG (2016). Ankle arthrodesis: a systematic approach and review of the literature. World J Orthop.

[6] Hughes JL, Weber H, Willenegger H, Kuner EH (1979). Evaluation of ankle fractures: non-operative and operative treatment. Clin Orthop Relat Res.

[7] Mosier-LaClair S, Pike H, Pomeroy G (2002). Syndesmosis injuries: acute, chronic, new techniques for failed management. Foot Ankle Clin.

[8] Pogliacomi F, De Filippo M, Casalini D (2021). Acute syndesmotic injuries in ankle fractures: from diagnosis to treatment and current concepts. World J Orthop.

[9] Thordarson DB, Samuelson M, Shepherd LE, Merkle PF, Lee J (2001). Bioabsorbable versus stainless steel screw fixation of the syndesmosis in pronation-lateral rotation ankle fractures: a prospective randomized trial. Foot Ankle Int.

[10] Ahmad J, Raikin SM, Pour AE, Haytmanek C (2009). Bioabsorbable screw fixation of the syndesmosis in unstable ankle injuries. Foot Ankle Int.

[11] Sinisaari IP, Lüthje PM, Mikkonen RH (2002). Ruptured tibio-fibular syndesmosis: comparison study of metallic to bioabsorbable fixation. Foot Ankle Int.

[12] Schepers T (2011). To retain or remove the syndesmotic screw: a review of literature. Arch Orthop Trauma Surg.

[13] van der Eng DM, Schep NW, Schepers T (2015). Bioabsorbable versus metallic screw fixation for tibiofibular syndesmotic ruptures: a meta-analysis. J Foot Ankle Surg.

[14] Stenquist DS, Ye MY, Kwon JY (2020). Acute and chronic syndesmotic instability: role of surgical stabilization. Clin Sports Med.

[15] Hovis WD, Kaiser BW, Watson JT, Bucholz RW (2002). Treatment of syndesmotic disruptions of the ankle with bioabsorbable screw fixation. J Bone Joint Surg Am.

[16] Gaiarsa GP, Dos Reis PR, Mattar R Jr, Silva Jdos S, Fernandez TD (2015). Comparative study between osteosynthesis with conventional and bioabsorbable implants in ankle fractures. Acta Ortop Bras.

[17] Kaukonen JP, Lamberg T, Korkala O, Pajarinen J (2005). Fixation of syndesmotic ruptures in 38 patients with a malleolar fracture: a randomized study comparing a metallic and a bioabsorbable screw. J Orthop Trauma.

[18] Sun H, Luo CF, Zhong B, Shi HP, Zhang CQ, Zeng BF (2014). A prospective, randomised trial comparing the use of absorbable and metallic screws in the fixation of distal tibiofibular syndesmosis injuries: mid-term follow-up. Bone Joint J.

[19] Vander Griend R, Savoie FH III, Hughes MS (2012). Cost considerations in orthopaedic surgery. Clin Orthop Relat Res.

[20] Sukotjo C, Lima-Neto TJ, Santiago Júnior JF, Faverani LP, Miloro M (2020). Is there a role for absorbable metals in surgery? A systematic review and meta-analysis of Mg/Mg alloy-based implants. Materials (Basel).

